# Reversal of Epigenetic Silencing Allows Robust HIV-1 Replication in the Absence of Integrase Function

**DOI:** 10.1128/mBio.01038-20

**Published:** 2020-06-02

**Authors:** Ishak D. Irwan, Heather L. Karnowski, Hal P. Bogerd, Kevin Tsai, Bryan R. Cullen

**Affiliations:** a Department of Molecular Genetics and Microbiology, Duke University Medical Center, Durham, North Carolina, USA; Johns Hopkins Bloomberg School of Public Health

**Keywords:** HIV-1, HTLV-1, Tax, integrase, cccDNA, integrase inhibitors, epigenetic gene regulation, NF-κB, human T-cell leukemia virus, human immunodeficiency virus

## Abstract

Integration of the proviral DNA intermediate into the host cell genome normally represents an essential step in the retroviral life cycle. While the reason(s) for this requirement remains unclear, it is known that unintegrated proviral DNA is epigenetically silenced. Here, we demonstrate that human immunodeficiency virus 1 (HIV-1) mutants lacking a functional integrase (IN) can mount a robust, spreading infection in cells expressing the Tax transcription factor encoded by human T-cell leukemia virus 1 (HTLV-1). In these cells, HIV-1 forms episomal DNA circles, analogous to hepatitis B virus (HBV) covalently closed circular DNAs (cccDNAs), that are transcriptionally active and fully capable of supporting viral replication. In the presence of Tax, induced NF-κB proteins are recruited to the long terminal repeat (LTR) promoters present on unintegrated HIV-1 DNA, and this recruitment in turn correlates with the loss of inhibitory epigenetic marks and the acquisition of activating marks on histones bound to viral DNA. Therefore, HIV-1 is capable of replication in the absence of integrase function if the epigenetic silencing of unintegrated viral DNA can be prevented or reversed.

## Abstract (precis)

While retroviral DNA is synthesized normally after infection by integrase-deficient viruses, the resultant episomal DNA is then epigenetically silenced. Here, we show that expression of the Tax transcription factor encoded by a second human retrovirus, HTLV-1, prevents or reverses the epigenetic silencing of unintegrated HIV-1 DNA and instead induces the addition of activating epigenetic marks and the recruitment of NF-κB/Rel proteins to the HIV-1 LTR promoter. Moreover, in the presence of Tax, the HIV-1 DNA circles that form in the absence of integrase function are not only efficiently transcribed but also support a spreading, pathogenic integrase-deficient (IN^−^) HIV-1 infection. Thus, retroviruses have the potential to replicate without integration, as is indeed seen with HBV. Moreover, these data suggest that integrase inhibitors may be less effective in the treatment of HIV-1 infections in individuals who are also coinfected with HTLV-1.

## INTRODUCTION

Integration of the proviral DNA intermediate into the chromosomal DNA of infected cells is a defining step of the retroviral life cycle (1, 2). Indeed, inhibition of integrase (IN) function is an effective means of blocking human immunodeficiency virus 1 (HIV-1) replication in not only T cells but also in macrophages, and IN inhibitors are routinely used as part of antiretroviral drug combinations (3–5). However, the reason(s) why proviral integration is essential for productive HIV-1 replication remain unclear. Of note, even though the hepadnaviruse hepatis B virus (HBV) also generates double-stranded DNA (dsDNA) copies of genome-length viral RNAs by reverse transcription, chromosomal integration of this dsDNA does not form part of their replication cycle. So why is HBV able to replicate without chromosomal integration, and, conversely, why is integration a critical step in all retroviral replication cycles?

A potential answer to these questions has emerged from the study of how not only HBV but also other DNA viruses, such as herpesviruses, are able to express their dsDNA genome. Specifically, HBx facilitates HBV gene expression by inducing the degradation of two cellular factors, SMC5 and SMC6, that otherwise block transcription of HBV episomes (6–8). SMC5 and SMC6 localize to PML nuclear bodies (PML-NBs), also called ND10, and depletion of the PML-NB components PML and Sp100 rescues the transcription of HBV episomes even in the absence of HBx (9). PML-NBs also serve as components of the innate immune response to infection by herpesviruses (10–12). In the case of herpes simplex virus 1 (HSV1), the incoming dsDNA genome is sensed by cellular factors, including IFI16, and then decorated with repressive chromatin modifications, including H3K9me^3^ and H3K27me^3^ (13–15). However, these inhibitory marks are then removed in a process that is dependent on the disruption of PML-NB function by the HSV1 immediate early protein ICP0 (13, 16). Thus, cells have evolved an innate antiviral restriction pathway that recognizes extrachromosomal viral dsDNA as “nonself” and induces its epigenetic silencing (14). Conversely, dsDNA viruses have evolved mechanisms to counter this restriction. In the case of HBV and most herpesviruses, this depends on viral proteins that induce the degradation of cellular factors that would otherwise epigenetically silence viral DNA, including SMC5/SMC6 in the case of HBV HBx and the PML-NB components PML and Sp100 in the case of HSV-1 ICP0 (6, 12). Conversely, we hypothesize that retroviruses avoid cellular restriction factors that recognize and silence extrachromosomal DNA by integrating their proviral DNA into the host genome, where it eludes detection. It is well established that unintegrated retroviral DNA is rapidly loaded with histones that are then decorated with repressive chromatin marks that induce epigenetic silencing (17, 18). In the case of unintegrated murine leukemia virus (MLV) DNA, epigenetic silencing is mediated not by PML-NBs but rather by a DNA binding protein called NP220 acting in concert with the human silencing hub (HUSH) complex (19). However, the HUSH complex does not play a role in silencing unintegrated HIV-1 DNA (19). Here, we report the surprising result that the epigenetic silencing of unintegrated HIV-1 DNA is prevented or reversed by ectopic expression of the Tax transcription factor encoded by human T-cell leukemia virus 1 (HTLV-1). In the presence of Tax, integrase-deficient (IN^−^) HIV-1 is able to effectively transcribe the circular DNA molecules that form in the nucleus in the absence of integrase function to mount a spreading cytopathic infection.

## RESULTS

**FIG 1:**
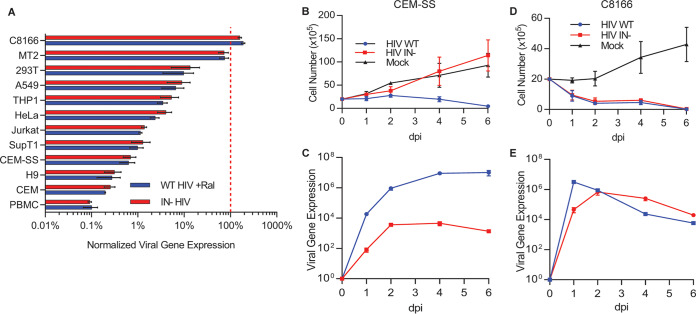
Differential gene expression and replication of integrase-deficient (IN^−^) HIV-1. (A) Nano luciferase (NLuc) activity from the indicated cell lines or activated peripheral blood mononuclear cells (PBMCs) infected with the wild type (WT), WT plus raltegravir (RAL), or with the D64V integrase mutant (IN^−^) NL4-3-based indicator virus in which the *nef* gene was replaced with the NLuc indicator gene (NL-NLuc) reporter virus at 48 hours postinfection (hpi). The cells used express CD4 naturally or artificially. NLuc expression levels were normalized to WT, set at 100%. *n* = 3, ± standard deviation (SD). Viable cell counts of (B) CEM-SS and (D) C8166 cells infected with WT or IN^−^ forms of the replication-competent NL-NLuc indicator virus. Virally encoded NLuc expression was measured for (C) CEM-SS and (E) C8166 cells infected with WT or IN^−^ NL-NLuc. All IN^−^ infections shown in panels B to E were conducted in the additional presence of 20 μM RAL.

If the inability of unintegrated HIV-1 DNA to be effectively transcribed is caused by host restriction factors, then the level of inhibition might vary between different cell types, as has indeed been previously reported (20). To confirm and extend this observation, we infected primary peripheral blood mononuclear cells (PBMCs) and various human cell lines with an NL4-3-based indicator virus in which the *nef* gene was replaced with the Nano luciferase (NLuc) indicator gene (NL-NLuc). Cells were infected with wild-type (WT) HIV-1, with an IN mutant (D64V) that lacks integrase function, or with WT HIV-1 in the presence of 20 μM raltegravir (RAL), which blocks IN function (21, 22). Levels of NLuc expression were quantified and normalized to WT HIV-1, which was set at 100%. Similar levels of NLuc expression were observed whether IN activity was blocked by the D64V mutation or by RAL (Fig. 1A). These data revealed variable levels of inhibition of HIV-1 gene expression when proviral integration was blocked. Thus, peripheral blood mononuclear cells (PBMCs), H9, CEM, CEM-SS, SupT1, and Jurkat cells all showed a >50-fold reduction in NLuc expression in the absence of IN function, while HeLa, THP1, A549, and 293T cells retained from 2% to 12% residual NLuc activity. Remarkably, MT2 cells retained ∼70% of the NLuc expression in the absence of IN function, while C8166 cells supported similar levels of NLuc expression whether IN was active or not (Fig. 1A). Moreover, while infection of CEM-SS cells with the D64V IN mutant resulted, as expected, in minimal viral replication (Fig. 1C) and did not reduce cell viability (Fig. 1B), IN^−^ HIV-1 was capable of almost WT levels of replication in C8166 cells (Fig. 1E), resulting in indistinguishable cytopathic effects (Fig. 1D).

**FIG 2:**
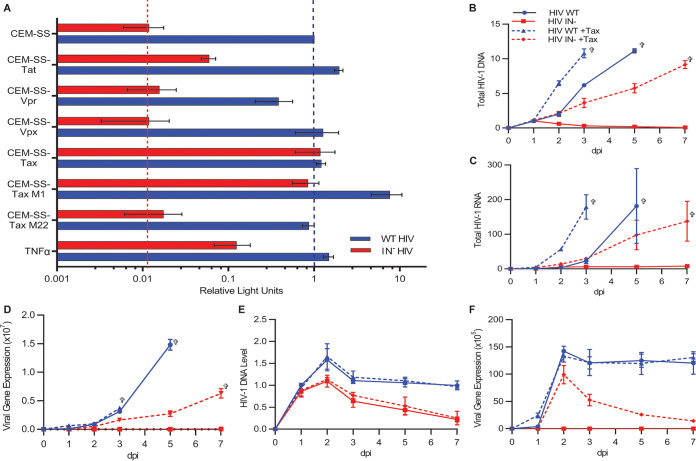
HTLV-1 Tax rescues integrase-defective HIV-1. (A) CEM-SS cells were transduced with tetracycline (Tet)-inducible lentiviral vectors expressing each of the indicated viral proteins. Expression was induced with 0.5 μg/ml doxycycline (Dox), and cells then infected with WT or IN^−^ NL-NLuc. NLuc levels were measured at 48 hpi. Tumor necrosis factor alpha (TNF-α; 1 ng/ml) was added at the time of infection. NLuc expression in each experiment was normalized to WT NL-NLuc-infected CEM-SS cells, which were set to 1. *n* = 3, ±SD. (B) Clonal CEM-SS cell lines with Tet-inducible Tax expression were infected with WT or IN^−^ NL-NLuc reporter virus with and without Dox induction. Total HIV-1 DNA levels in the cells were measured by quantitative PCR (qPCR) at the indicated times. (C and D) Results similar to those in panel B but quantifying changes in (C) total viral RNA, and (D) virally encoded NLuc expression over time. (E and F) Tet-inducible CEM-SS Tax cells were infected with nonspreading, VSV-G-pseudotyped, IN^+^ or IN^−^ NL-NLuc ΔEnv reporter virus in the presence and absence of Dox, and changes in (E) total DNA and (F) NLuc expression were quantified. All *y* axes show fold changes relative to WT HIV-1-infected, uninduced (without Dox or Tax) cells at day 1, which were set to 1; *n* = 3. All IN^−^ infections performed in the presence of 20 μM RAL. Crosses indicate last viable day of culture.

Both C8166 and MT2 cells are HTLV-1 infected and express the HTLV-1 Tax protein, which can activate transcription from the HIV-1 long terminal repeat (LTR) (23). To test whether Tax expression is sufficient to rescue the replication of IN^−^ HIV-1, we transduced CEM-SS T cells with a tetracycline (Tet)-inducible lentiviral vector expressing (i) Tax, the HIV-1 Tat transactivator, (ii) the HIV-1 Vpr protein, which has been reported to partially rescue gene expression from IN^−^ HIV-1 (24), (iii) HIV-2 Vpx, which was reported to block the inhibitory activity of the HUSH complex (25, 26), or (iv) the HIV-1 Tat protein. As shown in Fig. 2A, while the doxycycline (Dox)-induced expression of Tax fully rescued gene expression from IN^−^ HIV-1 virus, it only modestly increased WT HIV-1 gene expression. In contrast, induced overexpression of HIV-1 Tat had a small but nonspecific positive effect on gene expression from both WT and IN^−^ NL-NLuc, while induced expression of either HIV-1 Vpr or HIV-2 Vpx failed to affect either WT or IN^−^ HIV-1 (Fig. 2A). Importantly, all of these viral proteins were expressed at readily detectable levels after Dox addition (see Fig. S1A and S1B in the supplemental material), and only HIV-1 Vpr expression inhibited cell proliferation, as expected (see Fig. S2 in the supplemental material) (27, 28).

10.1128/mBio.01038-20.1 FIG S1 Dox-inducible expression of Tax but not Vpx, Vpr, or Tat rescues the expression of HIV-1 *gag* from unintegrated HIV-1 episomes. (A) Single-cell clones of CEM-SS cells transduced with a tetracycline (Tet)-inducible lentivector expressing HTLV-1 Tax or the indicated Tax mutants in the presence or absence of 0.5 μg/ml doxycycline (Dox). (B) Similarly to the experiment shown in panel A, cells were transduced with Tet-inducible lentivectors expressing HIV-2 Vpx, HIV-1 Vpr, or HIV-1 Tat. (C) Wild-type (WT) CEM-SS cells and Tet-inducible, Tax-expressing CEM-SS cells were infected with WT or integrase-deficient (IN^−^) HIV-1 in the presence or absence of Dox and probed on a Western blot for the indicated proteins. Download FIG S1, JPG file, 0.2 MB. Copyright © 2020 Irwan et al. 2020 Irwan et al. This content is distributed under the terms of the Creative Commons Attribution 4.0 International license.

10.1128/mBio.01038-20.2 FIG S2 Effect of Dox-induced expression of viral proteins on the growth of CEM-SS cells. Proliferation of WT CEM-SS cells, or of CEM-SS cells transduced with Dox-inducible lentiviral vectors encoding the indicated viral genes, in the presence and absence of Dox. Cell counts were performed on the indicated days postinduction (dpi) with 0.5 μg/ml doxycycline and in the absence of Dox. (A) HTLV-1 Tax, (B) M1 mutant Tax, (C) M22 mutant Tax, (D) HIV-2 Vpx, (E) HIV-1 Vpr, and (F) HIV-1 Tat. *n* = 3; results of a representative experiment are shown. Download FIG S2, JPG file, 0.5 MB. Copyright © 2020 Irwan et al. 2020 Irwan et al. This content is distributed under the terms of the Creative Commons Attribution 4.0 International license.

Tax activates both the NF-κB and CREB/cAMP response element (CRE) pathway in expressing cells, and Tax mutants lacking the ability to activate each of these pathways have been described (29). While the Tax M1 mutant, which lacks the ability to induce the CREB/CRE pathway, fully retained the ability to rescue IN^−^ HIV-1 gene expression, the Tax M22 mutant, which is unable to activate NF-κB, lost the ability to rescue IN^−^ HIV-1 gene expression, even though both mutants were expressed at similar levels (Fig. 2A and Fig. S1A). Further supporting a key role for NF-κB activation, treatment of WT CEM-SS cells with tumor necrosis factor alpha (TNF-α), a specific inducer of NF-κB function (30), also selectively rescued gene expression from the IN^−^ NL-NLuc vector, albeit not to the same level as did Tax (Fig. 2A).

### Tax expression allows IN^−^ HIV-1 to mount a spreading infection.

Given that induction of Tax expression in the otherwise nonpermissive CEM-SS T-cell line rescues IN^−^ HIV-1 gene expression (Fig. 2A), we asked whether Tax could also rescue IN^−^ HIV-1 replication. We analyzed the ability of WT or IN^−^ versions of the otherwise replication-competent NL-NLuc indicator virus to spread in CEM-SS cells transduced with the inducible Tax expression vector in the presence and absence of Dox. Parameters analyzed included total HIV-1 DNA levels (Fig. 2B), total HIV-1 RNA levels (Fig. 2C), and viral protein expression, as measured by analysis of the virally encoded NLuc protein (Fig. 1D) or of viral Gag expression (Fig. S1C). The IN^−^ form of the NL-NLuc virus failed to express significant levels of viral DNA, RNA, or protein in the absence of Tax, whereas in the presence of Tax, viral DNA, RNA, and protein were not only readily detected but increased over the course of the experiment, resulting in the death of the CEM-SS culture by 7 days postinfection (dpi) (Fig. 2B to D and Fig. S1C). To further confirm that Tax can indeed rescue the ability of IN^−^ HIV-1 to mount a spreading infection, we repeated this experiment using a mutant of the NL-NLuc indicator virus lacking a functional *env* gene (ΔEnv) that cannot spread. WT and IN^−^ forms of the NL-NLuc ΔEnv virus were pseudotyped with VSV-G and used to infect CEM-SS cells with and without Dox-induced Tax expression. As shown in Fig. 2E, the ΔEnv IN^−^ virus produced equal levels of HIV-1 DNA, regardless of Tax expression, that peaked at 2 dpi and then gradually declined to background levels by 7 dpi, as predicted if the viral DNA was unintegrated. In contrast, while the ΔEnv version of the IN^+^ virus also gave rise to peak DNA levels at 2 dpi, there was only a modest decline in viral DNA by 7 dpi, regardless of Tax expression. While Tax did not affect HIV-1 DNA levels in the context of a nonspreading infection, it did dramatically increase the level of viral gene expression from the IN^−^ form of the NL-NLuc ΔEnv virus, starting at 2 dpi. However, this positive effect was lost by 7 dpi, concomitant with the loss of unintegrated viral DNA (Fig. 2E and F).

**FIG 3:**
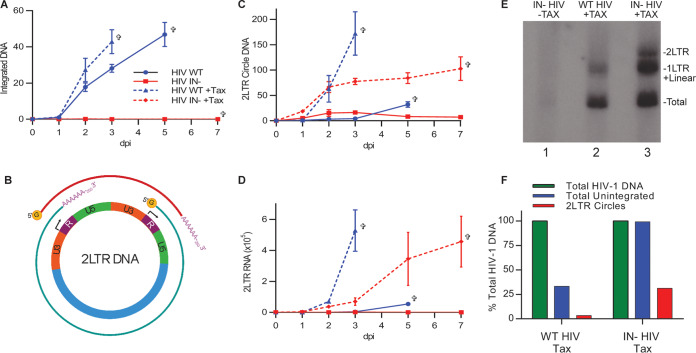
HTLV-1 Tax does not facilitate the illegitimate integration of IN^−^ HIV-1. (A) Quantification of integrated HIV-1 DNA in Tet-inducible CEM-SS Tax cells infected with WT or IN^−^ HIV-1, in the presence or absence of Tax induction, as measured by Alu-LTR qPCR. (B) Schematic showing the production of a short 2LTR transcript (red line) and the full-length transcript (green line) from circular 2LTR HIV-1 DNA. (C) Total 2LTR circle DNA was measured at the indicated time points by qPCR, using primers that span the U5-U3 junction, in CEM-SS Tax cells infected with WT or IN^−^ HIV-1 in the presence or absence of Tax. (D) Quantification of the predicted 2LTR RNA transcript in CEM-SS cells with or without Tax expression and infected with WT or IN^−^ HIV-1. All data in panels A, C, and D are normalized to WT-infected Tax-negative cells at day 1, set to 1; *n* = 3. Crosses indicate the last day viable cells were detected. (E) DNA from CEM-SS cells with or without Tax expression, infected with WT or IN^−^ HIV-1, was digested with MscI and XhoI and probed on a Southern blot with HIV-1 probes that detect specific DNA species that correspond to 2LTR DNA (3.4 kb), 1LTR plus linear unintegrated DNA (2.6 to 2.8 kb), and total HIV-1 DNA (1.9 kb). Note that lane 2 contains one-quarter of the amount of cellular DNA used in lanes 1 and 3. See Fig. S2 for details. (F) Quantification of the bands (2LTR+1LTR+linear and 2LTR) shown in panel E expressed as a percentage of total HIV-1 DNA, which was set at 100%.

To further confirm that Tax was not facilitating illegitimate HIV-1 DNA integration, we performed Alu-LTR quantitative PCR (qPCR) (31), which quantifies the level of integrated viral DNA in HIV-1-infected cultures. As shown in Fig. 3A, we observed high levels of integrated HIV-1 proviral DNA when the IN^+^ form of NL-NLuc was analyzed, while the IN^−^ form of NL-NLuc, as predicted, gave rise to undetectable levels of integrated proviral DNA regardless of Tax expression.

### Integrase-defective HIV-1 produces transcriptionally active DNA circles in the presence of Tax.

Unintegrated HIV-1 DNA can exist in three different forms in infected cell nuclei. These are linear DNA, the substrate for integration; 1LTR DNA circles, formed by homologous recombination of linear DNA; and 2LTR DNA circles, formed by nonhomologous end joining of linear DNA (32). While all three forms of unintegrated viral DNA could be transcriptionally active, 2LTR circles have the unique ability to generate a novel HIV-1 transcript, called 2LTR RNA, that initiates in what would normally be the 3′ LTR and is then polyadenylated in the 5′ LTR (Fig. 3B) (33). Analysis of the formation of 2LTR circular DNA in HIV-1 infected CEM-SS cells, in the presence and absence of Tax, revealed a low level in the IN− infected culture at early times after infection in the absence of Tax, but high and gradually increasing levels of 2LTR DNA in the presence of Tax (Fig. 3C). These data mirror what was seen for total viral DNA in IN^−^ virus-infected CEM-SS cells in the presence and absence of Tax (Fig. 2B). Interestingly, Tax had an unexpected effect on the formation of 2LTR circles by IN+ WT virus. Specifically, while WT HIV-1 growing in CEM-SS cells in the absence of Dox induction gave rise, as expected, to low levels of 2LTR circles, this level was dramatically increased when Tax expression was induced (Fig. 3C). Similarly, when we analyzed the expression level of the predicted 2LTR RNA by reverse transcription-quantitative PCR (qRT-PCR), we observed essentially undetectable levels in CEM-SS T cells infected by IN^−^ HIV-1 in the absence of Tax, but high and gradually increasing levels of 2LTR RNA when the same IN^−^ virus was used to infect Tax-expressing CEM-SS (Fig. 3D). Again, the WT IN^+^ version of the NL-NLuc indicator virus gave rise to low levels of 2LTR RNA in the absence of Tax, yet, in the presence of Tax, produced high levels of 2LTR RNA (Fig. 3D). Thus, Tax causes WT HIV-1 to form higher levels of 2LTR circular DNA, which is efficiently transcribed to generate both full-length HIV-1 transcripts and the 2LTR noncoding RNA (Fig. 3B and D). Arguably, these transcriptionally active circular HIV-1 DNA molecules represent functional homologs of the covalently closed circular DNAs (cccDNAs) that represent the transcriptionally active form of the HBV DNA genome.

To further confirm that Tax indeed induces a spreading infection due to the active transcription of unintegrated HIV-1 DNA circles, we performed a Southern blot analysis to quantify total viral DNA, 2LTR DNA and linear/1LTR DNA (these latter two forms cannot be distinguished; see Fig. S3 in the supplemental material for probe strategy). As shown in Fig. 3E, we again observed a dramatic increase in the level of HIV-1 DNA in Tax-expressing CEM-SS T cells infected with IN^−^ HIV-1 (lane 3) compared to that in the same virus in CEM-SS cells lacking Tax (lane 1). Moreover, compared to IN^+^ HIV-1 (lane 2), quantification revealed that ∼100% of the viral DNA detected in the IN^−^ infected culture was unintegrated, while ∼30% of the viral DNA was unintegrated in the Tax-expressing culture infected with IN^+^ virus (Fig. 3F). Even though Tax at least modestly inhibits HIV-1 DNA integration in cells infected with WT HIV-1 (Fig. 3C), we only observed low levels of 2LTR circular DNA in these cells. In contrast, 2LTR circles contributed ∼30% of the viral DNA detected in Tax-expressing CEM-SS cells infected with IN^−^ HIV-1 (Fig. 3F).

10.1128/mBio.01038-20.3 FIG S3 Southern blot restriction enzyme and probe binding schematic. DNA from HIV-1 infected cells was digested with MscI and XhoI (and DpnI to remove residual plasmid contamination) overnight. This cuts HIV-1 DNA at the indicated points, and these DNA fragments were then separated by gel electrophoresis. A P^32^ radiolabeled DNA probe that spans an MscI cut site was used to detect the DNA fragments (A to C) (approximate probe binding in purple). This probe detects a 1.9-kb DNA fragment that is released by all HIV-1 DNA forms (A to C), as well as a 2.6-kb fragment released by unintegrated linear HIV-1 DNA (A), a 2.8-kb fragment released by 1LTR circle DNA (B), and a 3.4-kb fragment released by 2LTR circle DNA (C). Integrated HIV-1 DNA only produces the 1.9-kb fragment. Download FIG S3, JPG file, 0.5 MB. Copyright © 2020 Irwan et al. 2020 Irwan et al. This content is distributed under the terms of the Creative Commons Attribution 4.0 International license.

### Tax expression prevents the epigenetic silencing of unintegrated, chromatinized HIV-1 DNA.

**FIG 4:**
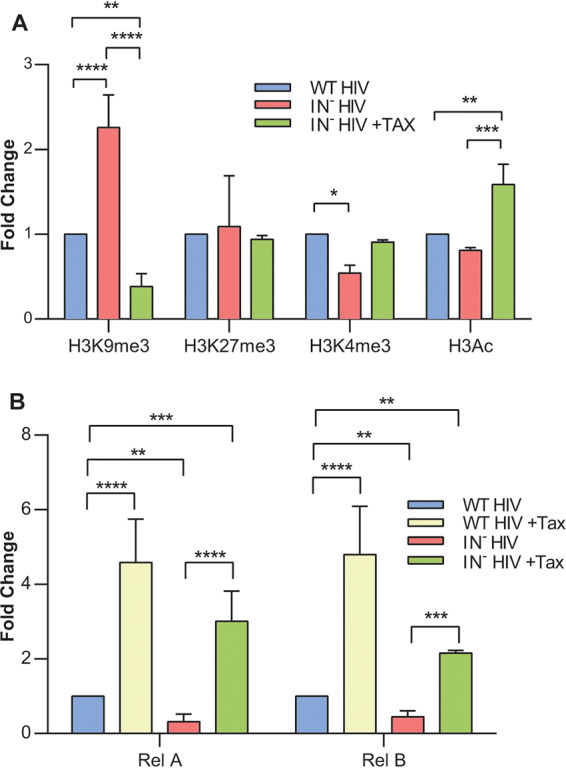
Tax changes the epigenetic state of viral DNA and Rel/NF-κB binding to the LTR. (A) CEM-SS cells in the presence or absence of Dox-induced Tax were infected with WT or IN^−^ NL-NLuc and subjected to chromatin immunoprecipitation (ChIP) using antibodies against the indicated histone modifications. The level of bound HIV-1 DNA was quantified by qPCR, with WT IN^+^ HIV-1 set at 1.0. Fold changes were normalized to total histone H3 levels. *n* = 3, ±SD. (B) Similarly to the experiment shown in panel A, NF-κB factors RelA and RelB were immunoprecipitated, and bound DNA was quantified by qPCR in CEM-SS cells with or without Dox-induced Tax expression and infected with IN^+^ or IN^−^ HIV-1. Cells were harvested at 2 dpi, and bound HIV-1 DNA was quantified using primers that amplify part of the viral LTR. Fold change normalized to WT IN^+^ HIV-1 in the absence of Tax, which was set at 1.0. *n* = 3, ±SD. *, *P* < 0.05; **, *P* < 0.01; ***, *P* < 0.001; ****, *P* < 0.0001 (2-way analysis of variance [ANOVA] with Tukey’s multiple-comparison test).

It has previously been reported that unintegrated HIV-1 DNA is rapidly loaded with core and linker histones and then epigenetically silenced due to the addition of inhibitory histone modifications (17). Therefore, we predicted that Tax, which induces the efficient transcription of unintegrated HIV-1 DNA, was likely preventing the latter effect. We used chromatin immunoprecipitation (ChIP)-PCR to quantify the level of two inhibitory chromatin modifications (H3K9me3 and H3K27me3) and two activating chromatin modifications (H3K4me3 and H3Ac) on integrated and unintegrated HIV-1 DNA in the presence and absence of Tax. As previously reported (17), unintegrated HIV-1 DNA was significantly enriched for repressive H3K9me3 and depleted for activating H3K4me3 compared to that in integrated proviruses (Fig. 4A). In contrast, in the presence of Tax, the H3K9me3 levels bound to unintegrated HIV-1 DNA were not only significantly lower than that seen with the same DNA in the absence of Tax but also lower than that seen on integrated HIV-1 DNA (Fig. 4A). Similarly, the level of activating H3Ac modifications was significantly higher on unintegrated HIV-1 DNA in the presence of Tax than that seen on either unintegrated or integrated HIV-1 DNA in the absence of Tax (Fig. 4A). We also observed a Tax-induced enhancement in the level of the activating H3K4me3 modification on unintegrated HIV-1 DNA, although this fell short of statistical significance (*P* = 0.059). Finally, neither integration nor Tax expression affected the level of H3K27me3 detected on viral DNA.

In Fig. 2A, we showed that the ability of Tax to rescue gene expression from unintegrated HIV-1 DNA not only required the ability to activate NF-κB but also that this effect could be partly mimicked by using TNF-α to activate NF-κB. Previously, Tax has been reported to activate both RelA/p65 and RelB (34), both of which are potent transcriptional activators that are known to bind the two canonical NF-κB sites found in the HIV-1 LTR U3 region. The use of ChIP-PCR to test whether Tax expression induced the recruitment of increased levels of RelA and/or RelB to the HIV-1 LTR indeed revealed a large, statistically significant increase in the recruitment of both RelA and RelB to both integrated and unintegrated HIV-1 DNA upon expression of Tax in infected CEM-SS cells (Fig. 4B).

**FIG 5:**
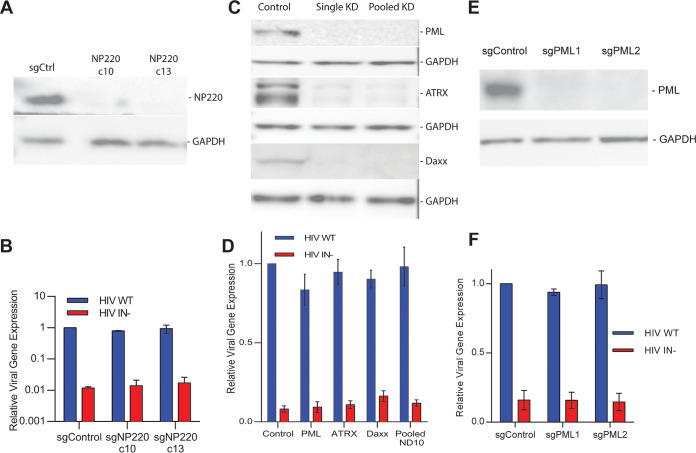
Neither NP220 nor PML-NBs appear to be involved in silencing unintegrated HIV-1 episomal DNA. (A) We generated two 293T-derived clonal cell lines, c10 and c13, in which expression of NP220 was blocked by CRISPR/Cas-mediated gene editing. Loss of detectable NP220 expression was confirmed by Western blotting. See Fig. S3 for details. (B) The NP220 knockout 293T clones c10 and c13 were infected with VSV-G-pseudotyped WT or IN^−^ NL-NLuc ΔEnv reporter virus. NLuc expression was determined at 48 h postinfection and is given normalized to the IN^+^ indicator virus in control cells, which was set at 1. (C) We used RNAi to knock down expression of the PML-NB components PML, ATRX, or Daxx either individually (“single KD”) or simultaneously (“pooled KD”) in CD4-expressing 293T cells, as visualized by Western blotting. (D) The PML-NB component knockdown cells from panel C were infected with WT or IN^−^ NL-NLuc virus, and NLuc expression was determined at 48 h postinfection. Data are normalized to control cells treated with an irrelevant small interfering RNA (siRNA) and infected with WT NL-NLuc, which was set at 1.0. (E) Two CD4-expressing 293T clones, sgPML1 and sgPML2, were generated by knockout of the key PML-NB component PML using CRISPR/Cas. Loss of PML expression was confirmed by Western blotting. See Fig. S3 for details. (F) The PML knockout 293T clones sgPML1 and sgPML2 were infected with WT or IN^−^ NL-NLuc virus, as described in panel B. NLuc expression was analyzed at 48 h postinfection and normalized to WT NL-NLuc in control cells, which was set at 1.0. For panels B, D, and F, *n* = 3, with SD indicated.

As noted above, it has previously been reported that the epigenetic silencing of unintegrated murine leukemia virus (MLV) proviral DNA is mediated by the cellular DNA binding protein NP220 acting in concert with the HUSH complex (19). Surprisingly, this report also documented that the HUSH complex was not involved in silencing unintegrated HIV-1 DNA, a result that is consistent with the inability of the HIV-2 Vpx protein, a known inhibitor of HUSH complex function (25, 26), to rescue gene expression from unintegrated HIV-1 DNA (Fig. 2A). We used CRISPR/Cas to mutationally inactivate the NP220 gene in 293T cells by deletion of the entire NP220 DNA binding domain and simultaneous introduction of stop codons (Fig. 5A; see also Fig. S4A and B in the supplemental material). Surprisingly, loss of NP220 function had no effect on the level of silencing of unintegrated HIV-1 DNA (Fig. 5B), thus confirming that the silencing of unintegrated MLV DNA and that of unintegrated HIV-1 DNA are indeed mechanistically distinct. The obvious other potential factor mediating unintegrated HIV-1 DNA silencing is PML-NBs, given their known role in the epigenetic silencing of nuclear DNA viruses (11). However, knockdown of the key PML-NB components PML, ATRX, and Daxx, either individually or simultaneously, using RNA interference (RNAi) did not significantly enhance gene expression from unintegrated HIV-1 DNA (Fig. 5C and D). It could be argued that RNAi does not fully deplete targeted proteins and that the residual level of PML-NB function might still be sufficient to silence unintegrated HIV-1 DNA. To address this issue, we used CRISPR/Cas to inactivate the *pml* gene in 293T cells by introduction of frameshift mutations (Fig. S4C). However, loss of PML expression (Fig. 5E) also failed to rescue gene expression from unintegrated HIV-1 proviruses (Fig. 5F). Therefore, the cellular mechanism that silences unintegrated HIV-1 DNA does not appear to involve either the HUSH complex or PML-NBs.

10.1128/mBio.01038-20.4 FIG S4 NP220 and PML knockout 293T cell clones were generated using CRISPR/Cas. (A) The NP220 DNA binding domain on exon 22 (highlighted in purple) was targeted for excision using two single guide RNAs (sgRNAs), highlighted in yellow and blue. The yellow/blue boundary is the predicted Cas9 cleavage site. (B) DNA sequences of clones of 293T cells transfected with the two sgRNAs showing excision of the NP220 DNA binding domain and the introduction of premature stop codons. (C) PML knockouts were generated by transfection of 293T cells with lentiCrisprv2 encoding either sgPML1 or sgPML2. Single-cell clones were isolated after puromycin selection, and the targeted region of the PML gene was PCR amplified, cloned and sequenced. PML clone 1 (sgPML1) has 10-, 19-, and 77-bp deletions, while PML clone 2 (sgPML2) has 7- and 11-bp deletions, all of which are predicted to introduce frameshift mutations. Download FIG S4, JPG file, 1.4 MB. Copyright © 2020 Irwan et al. 2020 Irwan et al. This content is distributed under the terms of the Creative Commons Attribution 4.0 International license.

## DISCUSSION

We report here the surprising finding that expression in T cells of the HTLV-1 transcription factor Tax allows integrase-deficient HIV-1 to mount a spreading, cytopathic infection characterized by increasing levels of HIV-1 DNA, RNA, and protein expression (Fig. 2). Increased HIV-1 DNA levels were not observed when a replication-incompetent, *env*-deficient HIV-1 strain was analyzed, and hence must result from virus spread (Fig. 2E and F). Interestingly, while all of the HIV-1 DNA produced during the spreading infection mounted by IN^−^ HIV-1 in the presence of Tax was, as expected, unintegrated (Fig. 3A and F), much of this DNA is in the form of HIV-1 DNA circles, with 2LTR circles contributing ∼30% of the total (Fig. 3E and F). These 2LTR circles in turn produce readily detectable levels of a unique HIV-1 noncoding RNA consisting solely of the two viral LTRs (Fig. 3B and D). Unexpectedly, the 2LTR RNA was also detected in Tax-expressing CEM-SS cells infected with IN^+^ HIV-1, but only minimally in non-Tax-expressing cells (Fig. 3D), which correlated with a substantial increase in the production of 2LTR circular DNA (Fig. 3C). The reason for this phenomenon is not known, but we speculate that Tax is activating cellular DNA binding proteins, including NF-κB (Fig. 4B), that bind to unintegrated HIV-1 DNA and not only induce the transcription of that DNA but also sterically hinder proviral integration, resulting in the increased production of transcriptionally active, unintegrated HIV-1 DNA in IN^+^ HIV-1 infected cells.

Previously, it was reported that unintegrated retroviral DNA is rapidly chromatinized after nuclear entry and then decorated with inhibitory epigenetic marks that are removed by an unknown process after integration occurs (17, 18). Here, we demonstrate that Tax expression also reduces the deposition of inhibitory chromatin marks, and increases the deposition of activating marks, on unintegrated proviral DNA (Fig. 4A). This, in turn, correlates with an increase in the recruitment of the NF-κB/Rel family members RelA/p65 and RelB, both of which are activated by Tax, to the HIV-1 LTR on unintegrated DNA (Fig. 4B). Because activation of NF-κB is essential for the ability of Tax to rescue the viability of IN^−^ HIV-1 (Fig. 2A), and because treatment with the NF-κB inducer TNF-α also selectively rescues gene expression from IN^−^ HIV-1 (Fig. 2A), we speculate that NF-κB recruitment to the unintegrated viral DNA induces the observed epigenetic changes. However, Tax has also been proposed to play a role in chromatin remodeling in HTLV-1-infected cells (34, 35). We observed that Tax also enhances the recruitment of NF-κB proteins to integrated HIV-1 DNA, as previously reported (23), which is consistent with the higher level of IN^+^ HIV-1 gene expression shown in, for example, Fig. 2C.

Our data, and previous work from the Goff laboratory, are consistent with the hypothesis that proviral DNA integration allows escape from the epigenetic silencing that occurs if integration is blocked (18). However, this hypothesis does not preclude the possibility that integration also facilitates other aspects of retroviral replication. For example, integrated proviruses have the potential to be passed on to daughter cells after cell division, and some retroviruses, such as HTLV-I, clearly use vertical transmission as a mechanism to increase viral load. In addition, in rapidly dividing cells, such as activated T cells, unintegrated HIV-1 proviruses, which lack an origin of replication, are rapidly lost (Fig. 2E). However, IN inhibitors also block HIV-1 gene expression in nondividing macrophages (4, 36), so this cannot be the only reason for proviral integration.

Given that many nuclear DNA viruses express proteins, such as HSV-1 ICP0 and the HBV HBx protein, that block the epigenetic silencing of their episomal DNA genomes, the question arises as to why retroviruses have not evolved a similar strategy. One answer is that proviral integration clearly serves several purposes, as noted above, but the second is that something similar may in fact have occurred. If a simple retrovirus were to somehow acquire a gene product that could block epigenetic silencing of unintegrated proviruses, then one might expect the resultant virus to retain the *gag* and *env* genes, as well as a truncated *pol* gene lacking integrase, and to acquire a novel noncoding gene that blocks epigenetic silencing. In fact, this is a reasonable description of the HBV genome (37). HBV encodes a Gag homolog called the core (C) protein, Env homologs called surface (S) proteins, a Pol homolog called polymerase (P) protein that retains reverse transcriptase activity yet lacks integrase function, and finally the HBx nonstructural protein that blocks the epigenetic silencing of viral cccDNAs (9).

Two papers from the Engelman laboratory are relevant to the findings reported here. Specifically, Nakajima et al. (20) reported that IN^−^ HIV-1 can replicate in the HTLV-1-infected T-cell lines C8166 and MT4 but not in T-cell lines not infected by HTLV-1, such as Jurkat. These authors concluded that “productive replication in the absence of integrase function most likely required the illegitimate integration of HIV-1 into host chromosomes” and, as a result, they did not investigate whether Tax expression was indeed either necessary or sufficient to support IN^−^ HIV-1 replication, as we show here. Moreover, our data demonstrate that IN^−^ HIV-1 replication clearly does not involve “illegitimate integration” (Fig. 3) but rather results from the active transcription of unintegrated HIV-1 DNA. In a second paper from the Engelman laboratory, it was demonstrated that, if the HIV-1 genome was modified by insertion of an origin of DNA replication from the polyomavirus simian virus 40 (SV40) and engineered to express SV40 T antigen (TAg), then IN^−^ HIV-1 replication was possible (38). As one would predict, this rescue correlated with the amplification of unintegrated HIV-1 DNA circles in the presence of TAg. In contrast, rescue of IN^−^ HIV-1 replication by Tax does not result in any increase in HIV-1 DNA copy numbers except that due to virus spread, as seen most clearly when viral replication is blocked after one round (Fig. 2E).

Over 10 million people worldwide are infected with HTLV-1, and HTLV-1 infection is widespread in certain populations across the world. Indeed, rates of HTLV-1/HIV-1 coinfection of up to 10% have been reported in some countries in Africa (39). HTLV-1, like HIV-1, infects T lymphocytes, but unlike HIV-1, which rapidly kills infected T cells, HTLV-1 Tax activates cellular survival and proliferative pathways that instead induce T-cell proliferation, eventually leading, in a minority of infected patients, to an aggressive cancer called adult T-cell leukemia (34, 40). HTLV-1-infected CD4-positive (CD4^+^) T cells can be fairly common in infected individuals, and the data reported here demonstrate that Tax expression can rescue the replication of IN^−^ HIV-1 regardless of whether integrase function is lost due to mutagenesis or drug treatment (Fig. 1 and 2). We therefore speculate that integrase inhibitors may be less effective in the treatment of dually HTLV-1- and HIV-1-infected individuals.

## MATERIALS AND METHODS

### Cell culture and cell line generation.

293T, HeLa, and A549 cells were cultured in Dulbecco’s modified Eagle’s medium (DMEM) supplemented with 10% fetal bovine serum (FBS) and antibiotic-antimycotic solution. CEM, CEM-SS, H9, SupT1, MT2, C8166, and THP-1 cells were cultured in Roswell Park Memorial Institute (RPMI) medium supplemented with 10% FBS and antibiotic-antimycotic. Primary CD4^+^ T cells were cultured in RPMI medium with 10% FBS and antibiotic-antimycotic and supplemented with interleukin 2 (IL-2) 72 h before infection. CD4^+^ 293T, HeLa, and A549 cells were generated by transducing cells with a retroviral vector expressing CD4 (pBabe-CD4) (41), then selecting for puromycin resistance (1 μg/ml). Cells were then single-cell cloned and screened for CD4 expression.

All Tet-inducible viral proteins were made in the lentiviral pTREX vector (42). HTLV-1 Tax, HIV-2 Vpx, HIV-1 Tat and HIV-1 Vpr were PCR amplified, cloned into pTREX and sequence verified. The M1 and M22 Tax mutants were created by mutating WT Tax (29) using overlap-extension PCR to introduce the H3S (M1) or G137A/L138S (M22) mutations. Lentiviruses were packaged by transfecting 5 × 10^6^ 293T cells in a 15-cm dish with 15 μg of the lentiviral vector, as well as 10 μg and 5 μg of the packaging plasmids pCMVR8.74 and pMD2.G, respectively, using polyethylenimine (PEI). The medium was changed 24 h posttransfection (hpt), and supernatants were collected at 72 hpt, filtered through a 0.45-μm filter, and run through a 100,000-molecular weight cutoff (MWCO) concentrator (Amicon). CEM-SS cells (5 × 10^6^) were incubated overnight with 2 ml of the concentrated supernatant at 37°C. The medium was then replaced with fresh RPMI medium, and cells were incubated for 48 h. At this point, the medium was replaced with fresh RPMI medium supplemented with 1 μg/ml of puromycin to select for transduced cells. Cells were tested for Tet-inducible expression of viral proteins by Western blotting using the antibodies against Tax (29), Vpx (catalog no. 2710; NIH AIDS Reagent) (43), Vpr (catalog no. 51143-1-AP; Proteintech), and Tat (44). Gag monoclonal antibody 24-3 was described previously (45).

### Purification of CD4^+^ T cells from PBMCs.

PBMCs were isolated from total blood by density gradient centrifugation (lymphocyte separation medium, no. 25–072-CV; Cellgro). CD4^+^ T cells were isolated using the Dynabead CD4-positive isolation kit (catalog no, 1131D; Invitrogen), then activated by incubation in phytohemagglutinin (PHA) and mouse monoclonal antibodies specific for human CD28 and CD49d (catalog no. 347690; BD Biosciences) for 3 days (46).

### HIV-1 production.

In the pNL-NLuc reporter virus used in spreading infections, the viral *nef* gene in NL4-3 is replaced with the NLuc indicator gene (47). Nonspreading infections were carried out using similar indicator viruses bearing a 943-bp deletion in *env* and either NLuc (NL-NLuc ΔEnv) or GFP (NL-GFP ΔEnv) in place of *nef*. These viruses expressed either WT integrase or the D64V (IN^−^) mutant. Plasmids expressing the replication-competent NL-NLuc provirus or the nonspreading ΔEnv proviruses were transfected into 293T using PEI. Nonspreading ΔEnv proviruses were cotransfected into 293T cells along with the pMD2.G plasmid encoding VSV-G. After 24 h, spent medium was replaced with fresh medium. At 72 hpt, supernatant medium was filtered through a 0.45-μm filter. WT or IN^−^ HIV-1-containing supernatant media were normalized by p24 levels measured by enzyme-limited immunosorbent assay (ELISA), then used to infect target cells. Cells were washed three times in PBS, lysed in passive lysis buffer (Promega), and assayed for NLuc activity using the Nano-Glo luciferase assay on a Lumat LB9507 luminometer (Berthold Technologies).

### Analysis of HIV-1 replication and expression in Tet-inducible Tax cells.

Tet-inducible HTLV-1 Tax CEM-SS cells (10^7^) were resuspended in 10 ml of RPMI medium and infected with either WT or IN^−^ NL-NLuc virus in the presence or absence of 0.5 μg/ml doxycycline (Sigma). Viral supernatants were pretreated with 5 U/ml DNase I for 1 h at 37°C to remove residual plasmid DNA. All IN^−^ HIV-1 infections were supplemented with 20 μM raltegravir to prevent revertant mutations. Live cells (10^6^) were harvested at 1, 2, 3, 5, and 7 dpi and aliquoted to assay NLuc and for DNA and RNA extraction. Full retention of the D64V mutation was confirmed by DNA sequencing at the end of all IN^−^ virus growth experiments.

For DNA analysis, cells were pelleted and washed three times in cold PBS and then incubated with DpnI (NEB) to remove any residual plasmid contamination. DNA was then extracted using DNA Miniprep Plus columns (Zymo Research) according to the manufacturer’s instructions. For RNA analysis, cells were lysed in TRIzol (Thermo Fisher Scientific), and RNA was harvested according to the manufacturer’s instructions and DNase I treated overnight. The RNA was then converted to cDNA using a high-capacity cDNA reverse transcription kit (Applied Biosystems).

All quantitative PCRs were performed in triplicate in a StepOnePlus or QuantStudio 3 real-time PCR system according to the manufacturer’s instructions. Relative quantification of HIV-1 DNA levels was performed using the threshold cycle (ΔΔ*C_T_*) method with β-actin as an internal control (48). For experiments analyzing total HIV-1 DNA and RNA levels, HIV-1 DNA/cDNA was PCR amplified with a custom total HIV-1 TaqMan primer set that amplifies the U5-*gag* region. β-Actin DNA was PCR amplified using a premade TaqMan primer set, while β-actin cDNA in RNA analyses was quantified using a separate TaqMan primer set amplifying across a β-actin splice junction. 2LTR DNA and RNA were similarly quantified using TaqMan probes that amplify across the U5-U3 junction (49).

For Alu-LTR real-time nested qPCR, DNA was amplified using a modified version of the nested PCR approach described previously (31). Briefly, an initial nonsaturating PCR using the primers ALU1, ALU2, and L-HIV was performed using DNA isolated from HIV-1-infected cells. After the PCR products were purified using a PCR Kleen kit (Bio-Rad), nested qPCR was performed using the primers AA55M and L and the SYBR green master mix (Thermo Fisher Scientific). All qPCR primer sequences used to quantify total, 2LTR, and Alu-LTR viral DNA/RNA are shown in Table S1 in the supplemental material.

10.1128/mBio.01038-20.5 TABLE S1 Oligonucleotide sequences. Download Table S1, DOCX file, 0.01 MB. Copyright © 2020 Irwan et al. 2020 Irwan et al. This content is distributed under the terms of the Creative Commons Attribution 4.0 International license.

### Small interfering RNA knockdowns and single guide RNA knockouts.

CD4^+^ 293T cells were transfected with 25 pmol of pooled small interfering RNA (siRNA) (3 siRNA per pool; Origene) with RNAiMax (Thermo Fisher) twice (on day 1 and 3) and infected on day 4 with WT or IN^−^ NL-NLuc virus. Infected cells were assayed for NLuc activity at 2 dpi. Knockdowns were confirmed by Western blotting using antibodies against PML (catalog no. A301-167A-M; Bethyl), ATRX (catalog no. HPA001906; Sigma), and Daxx (catalog no. D7810; Sigma).

293T cells were transfected with pLentiCrispr v2 (39) encoding the relevant single guide RNA (sgRNA) sequences using PEI (Table S1). Cells were selected for puromycin resistance 2 days posttransfection, then single-cell cloned.

The area around the NP220 sgRNA cut sites in the single cell clones was amplified using primers NP220_NBD_FP and NP220_NBD_RP, cloned then sequenced to detect the expected deletion. The expression of NP220 in the selected clones was also assayed by Western blotting (catalog no. A301-548A-M; Bethyl). Cells were infected with VSV-G pseudotyped IN^+^ or IN^−^ NL-NLuc ΔEnv reporter virus and assayed for NLuc activity at 2 dpi.

Similarly, sgPML single-cell clones were assayed for PML expression by Western blotting (catalog no. A301-167A-M; Bethyl). DNA from PML-nonexpressing cells was extracted, and the region of interest around the cut site was amplified, cloned, and sequenced.

### Western blot analyses.

Cells were harvested and lysed in Laemmli buffer, sonicated, and denatured at 95°C for 15 min. Lysates were subjected to electrophoresis on 4 to 20% SDS-polyacrylamide gels (Bio-Rad), transferred onto nitrocellulose membranes, and then blocked in 5% milk in PBS plus 0.1% Tween. Membranes were incubated in primary and either an anti-mouse horseradish peroxidase (HRP) secondary antibody (catalog no. A9044; Sigma), or an anti-rabbit HRP antibody (catalog no. A6154; Sigma) diluted in 5% milk in PBS plus 0.1% Tween for 1 h each and then washed in PBS plus 0.1% Tween. The membranes were incubated with a luminol-based enhanced chemiluminescent (ECL) substrate and signals were visualized using GeneSnap (Syngene).

### Southern blot analysis.

Southern blot analyses were performed as described previously (50). Briefly, DNA was extracted from CEM-SS or Tet-inducible Tax CEM-SS cells infected with DNase I-treated WT or IN^−^ HIV-1 at 2 dpi using a DNA Miniprep Plus kit (Zymo Research). Purified DNA was digested with MscI, XhoI, and DpnI (NEB) overnight and recovered by ethanol precipitation, then 10 μg of DNA was run on a 1% Tris-borate-EDTA (TBE) gel. The gel was soaked in denaturation solution (1.5 M NaCl and 0.5 M NaOH), then washed in neutralization buffer (3 M NaCl and 0.5 M Tris-HCl [pH 7.0]). DNA was transferred overnight onto a GeneScreen Plus nylon membrane (PerkinElmer) by capillary action, the membrane washed in 2× SSC (1× SSC is 0.15 M NaCl plus 0.015 M sodium citrate), then UV-cross-linked in a Stratalinker 2400 at 3,000 μJ power (Stratagene). The membrane was then blocked in ExpressHyb hybridization solution (Clontech) for 1 h.

HIV-1-specific DNA was amplified by PCR from the pNL4-3 plasmid using the primers 5′-AGAAGAAATGATGACAGCATG-3′ and 5′-TGCCAGTTCTAGCTCTG-3′. The radiolabeled probe was generated using the Prime-a-Gene labeling system (Promega) and ^32^P-dCTP (Perkin Elmer), then denatured and hybridized onto the blocked membrane overnight at 60°C. The membrane was then washed before bands were visualized on film and quantified using a Typhoon phosphorimager (Amersham).

### ChIP-qPCR.

Tet-inducible Tax CEM-SS cells (10^7^) were resuspended in 10 ml of medium and infected with either WT or IN^−^ HIV-1 virus. At 48 hpi, cells were rinsed twice with PBS and cross-linked with 1% formaldehyde for 20 min at 25°C, quenched in 0.125 M glycine for 5 min, and lysed in ChIP lysis buffer (50 mM Tris-HCl [pH 8.0], 1% sodium dodecyl sulfate, and 10 mM EDTA). Cell lysates were then sonicated with a Fisher Sonic Dismembrator 60 (output, 4.5; 20-sec pulse repeated 6 times on ice with 40 sec between each sonication). The supernatant containing sonicated chromatin was precleared by the addition of magnetic Protein G Dynabeads (Thermo Fisher) that had been pretreated with denatured salmon sperm DNA (Invitrogen). The magnetic beads were removed, and the sonicated chromatin was immunoprecipitated overnight at 4°C using 2.5 μg of the indicated antibody in ChIP dilution buffer (16.7 mM Tris-HCl [pH 8.0], 1% Triton X-100, 0.01% SDS, 150 mM NaCl, and 1.2 mM EDTA). Anti-H3K4me3, H3K27me3, and H3Ac antibodies were obtained from EMD Millipore (catalog no. 07-473, 07-449, and 06-599), while p65 (RelA) and RelB antibodies were obtained from Santa Cruz (catalog no. 8008 and 48366). H3K9me3 (catalog no. 39161; Active Motif), total H3 (catalog no. ab1791; Abcam), and IgG (catalog no. 2729S; Cell Signaling) antibodies were obtained from the indicated suppliers.

Five percent of the sonicated chromatin was stored as input DNA without further treatment until the reverse cross-linking step. The next day, the incubated chromatin-antibody mixture was incubated with the pretreated Dynabeads for 2 h at 4°C, and then washed with ChIP low-salt buffer (20 mM Tris-HCl [pH 8.0], 1% Triton X-100, 0.1% SDS, 150 mM NaCl, and 2 mM EDTA), ChIP high-salt buffer (20 mM Tris-HCl [pH 8.0], 1% Triton X-100, 0.1% SDS, 500 mM NaCl, and 2 mM EDTA), ChIP LiCl buffer (10 mM Tris-HCl [pH 8.0], 1% NP-40, 250 mM LiCl, 1 mM EDTA, and 1% Na-deoxycholate) and Tris-EDTA (TE) buffer (10 mM Tris-HCl [pH 8.0] and 1 mM EDTA). Protein-DNA complexes were eluted from the beads with an elution buffer (0.1 M NaHCO_3_ and 1% SDS), then de-cross-linked by incubating at 65°C for 16 h and 95°C for 15 min, and then proteins were removed by adding proteinase K at 50°C for 3 h. DNA was then purified using a DNA Miniprep Plus kit (Zymo) and digested with DpnI (NEB) to remove any plasmid contamination before being used for qPCR analysis using primers that amplify U5-R on HIV-1 and the SYBR green master mix (Thermo Fisher Scientific). ΔΔ*C_T_* was calculated relative to total histone H3 levels.
