# Supplementary figures and images for: Reversal of Epigenetic Silencing Allows Robust HIV-1 Replication in the Absence of Integrase Function
Source: mBio. 2020 Jun 2;11(3):e01038-20. doi: 10.1128/mBio.01038-20 (PMC7267885; doi:10.1128/mBio.01038-20)

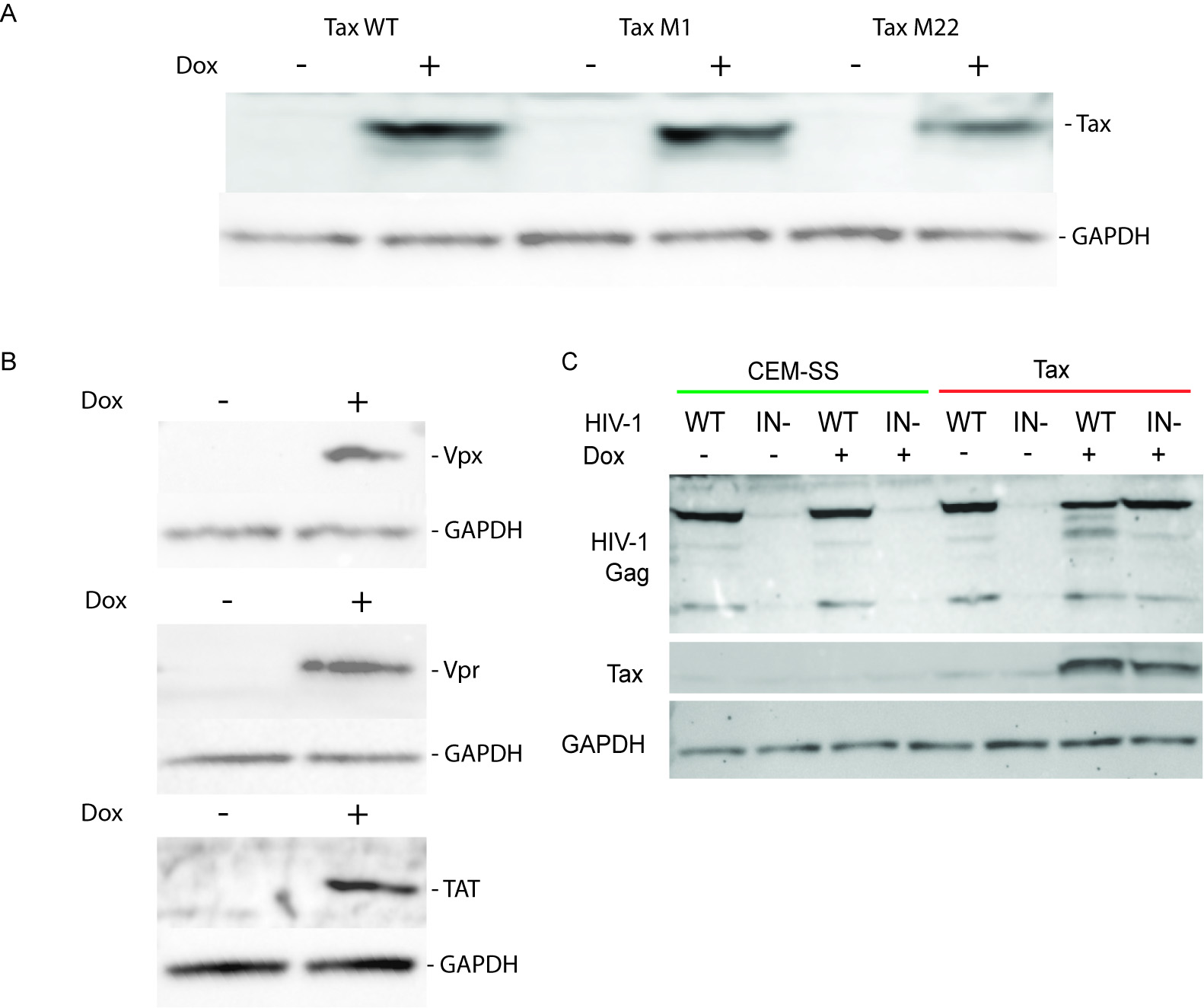

Supplement: FIG S1 [file mBio.01038-20-sf001.jpg]

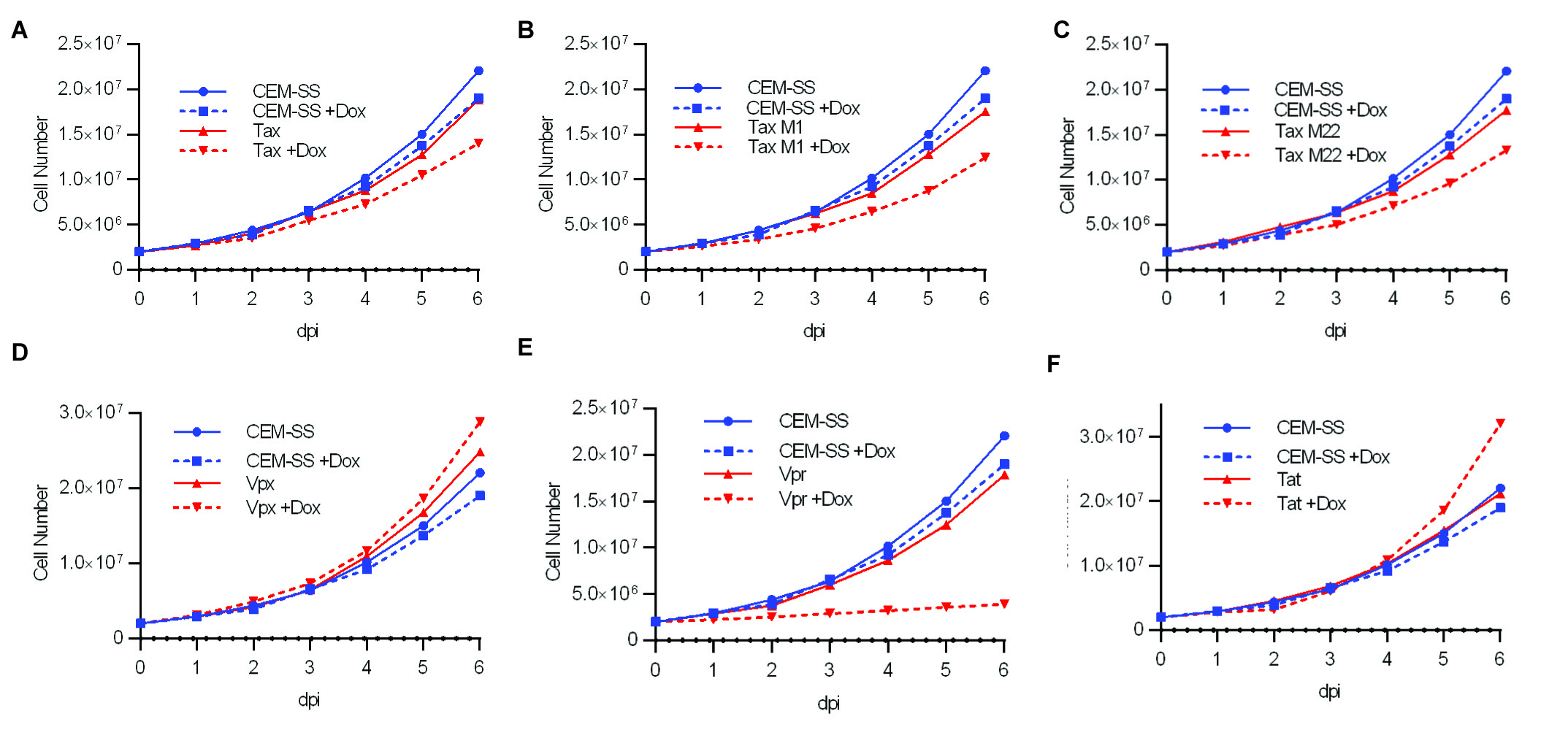

Supplement: FIG S2 [file mBio.01038-20-sf002.jpg]

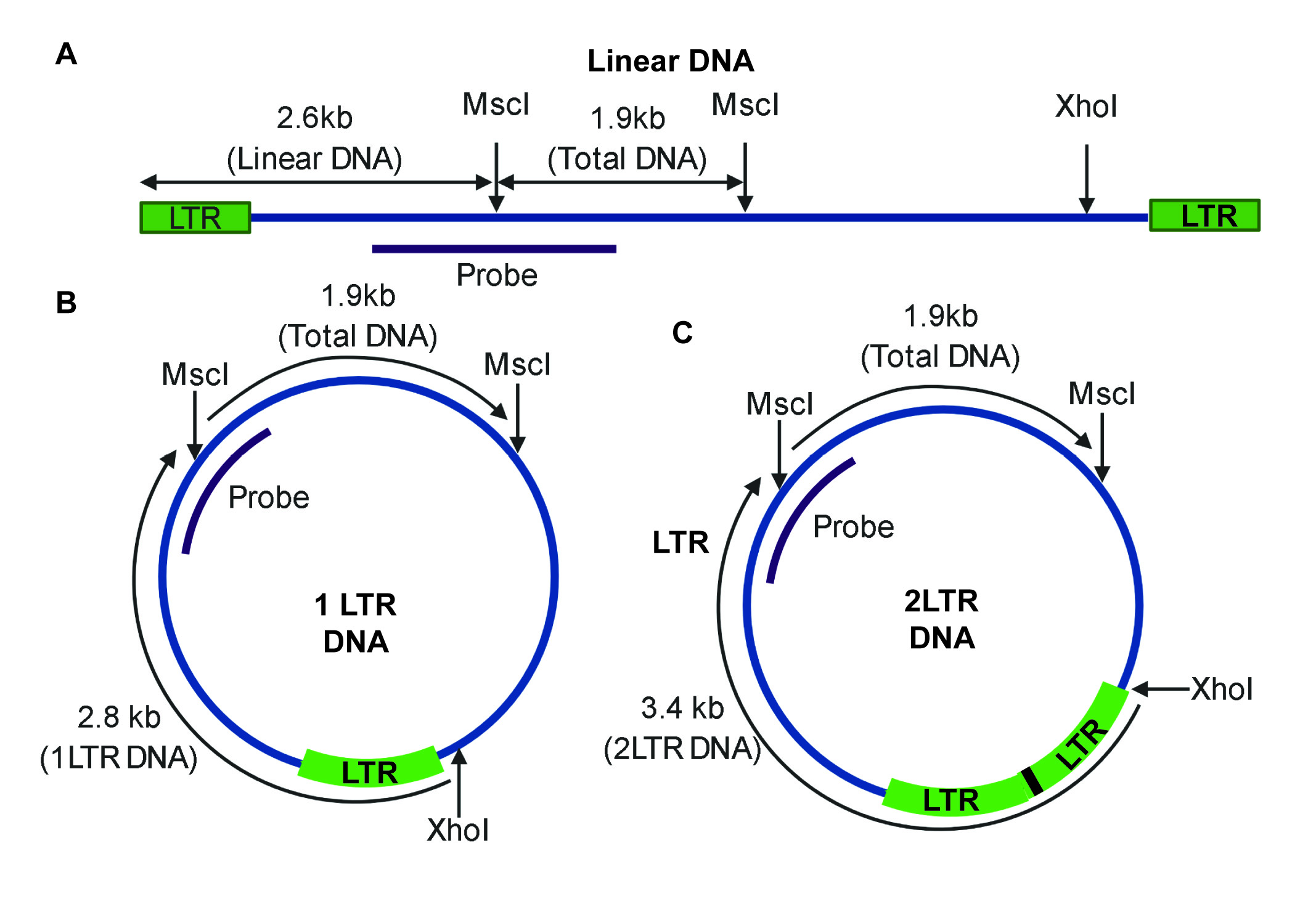

Supplement: FIG S3 [file mBio.01038-20-sf003.jpg]

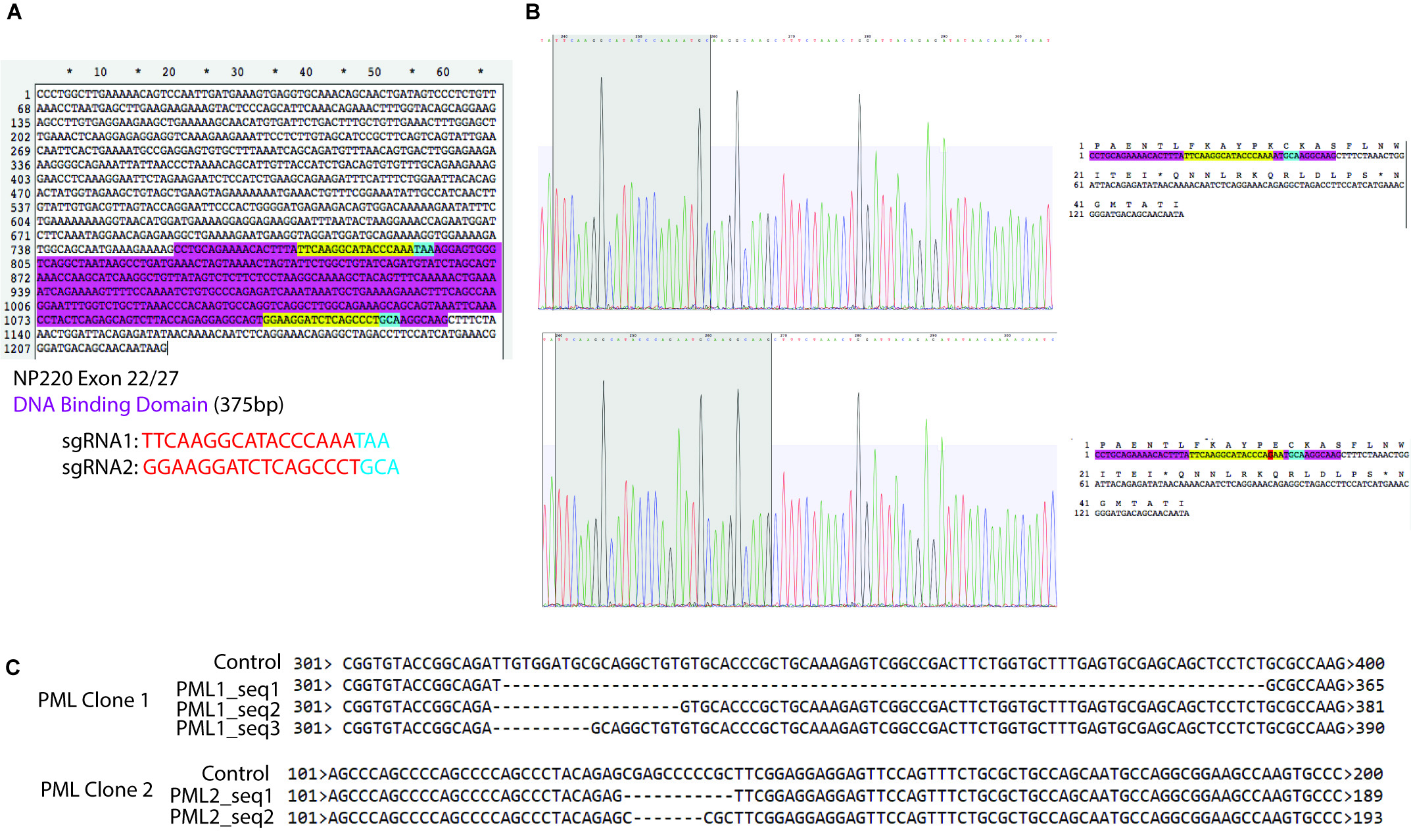

Supplement: FIG S4 [file mBio.01038-20-sf004.jpg]
