# Supplementary material for: Reversal of Epigenetic Silencing Allows Robust HIV-1 Replication in the Absence of Integrase Function
Source: mBio. 2020 Jun 2;11(3):e01038-20. doi: 10.1128/mBio.01038-20 (PMC7267885; doi:10.1128/mBio.01038-20)
Supplement: TABLE S1 [file mBio.01038-20-st001.docx]

**Table S1. Oligonucleotide sequences.**

| Oligo Name | Sequence (5’-3’) |
| --- | --- |
| sgNP220_DNABDg1_Top | CACCGTTCAAGGCATACCCAAATAA |
| sgNP220_DNABDg1_Bottom | AAACTTATTTGGGTATGCCTTGAAC |
| sgNP220_DNABDg2_Top | CACCGGGAAGGATCTCAGCCCTGCA |
| sgNP220_DNABDg2_Bottom | AAACTGCAGGGCTGAGATCCTTCCC |
| NP220_DNABD_FP | TGTGTCGACAGGTAACATGGATGAAAAGGAGGA |
| NP220_DNABD_RP | TGTGCTAGCACTGTGCCATTCTTCCTACCC |
| sgPML1_Top | CACCGCAATCTGCCGGTACACCGAC |
| sgPML1_Bottom | AAACGTCGGTGTACCGGCAGATTGC |
| sgPML2_Top | CACCGGGAACTCCTCCTCCGAAGCG |
| sgPML2_Bottom | AAACCGCTTCGGAGGAGGAGTTCCC |
| 2LTR_FP | AACTAGGGAACCCACTGCTTAAG |
| 2LTR_RP | TCCACAGATCAAGGATATCTTGTC |
| 2LTR_probe | FAM-ACACTACTTGAAGCACTCAAGGCAAGCTTT-TAMRA |
| TotalHIV_FP | TGTGTGCCCGTCTGTTGTGT |
| TotalHIV_RP | GAGTCCTGCGTCGAGAGAGC |
| TotalHIV_probe | FAM-CAGTGGCGCCCGAACAGGGA-TAMRA |
| Actin_DNA_FP | TGGACTTCGAGCAAGAGATG |
| Actin_DNA_RP | GAAGGAAGGCTGGAAGAGTG |
| Actin_DNA_probe | FAM-CGGCTGCTTCCAGCTCCTCC-TAMRA |
| Actin_RNA_spliced_FP | CAATGAAGATCAAG ATCATTGC |
| Actin_RNA_spliced_RP | AAGCATTTGCGGTGGAC |
| Actin_RNA_spliced_probe | FAM-TCCACCTTCCAGCAGATGTGGATCAGCAAG-TAMRA |
| HIV_R_FP | CTCTCTGGTTAGACCAGATC |
| HIV_U5_RP | GCTAGAGATTTTCCACACTG |
| Alu1 | TCCCAGCTACTGGGGAGGCTGAGG |
| Alu2 | GCCTCCCAAAGTGCTGGGATTACA |
| L-HIV | ATGCCACGTAAGCGAAACTTAAGCCTCAATAAAGCTTGC |
| L | ATGCCACGTAAGCGAAAC |
| AA55M | GCTAGAGATTTTCCACACTGACTAA |
| Southern_HIV_FP | AGAAGAAATGATGACAGCATG |
| Southern_HIV_RP | TGCCAGTTCTAGCTCTG |
